# Osteoblastic Cytokine Response to Gray and White Mineral Trioxide Aggregate 

**Published:** 2011-08-15

**Authors:** Maryam Bidar, Mohammad Hasan Zarrabi, Jalil Tavakol Afshari, Navid Aghasizadeh, Neda Naghavi, Maryam Forghanirad, Niloufar Attaran

**Affiliations:** 1 *Department of Endodontics, Dental Research Center/Mashad University of Medical Sciences, Mashad, Iran.*; 2 *Department of Immunology, Dental School, Mashad University of Medical Sciences, Mashad, Iran.*; 3 *Endodontis, Mashad, Iran.*; 4 *Dental Student, Gilan University of Medical Sciences, Rasht, Iran.*

**Keywords:** Biocompatibility, Human, Osteoblast, Interleukin-1β, IRMCement, Mineral Trioxide Aggregate

## Abstract

**INTRODUCTION:**The materials used for root-end filling and perforation repair are in direct contact with live tissues *e.g.* bone and connective tissue; their effects however, are uncertain. The aim of this *ex vivo*study was to evaluate the osteoblastic secretory activity adjacent to gray and white mineral trioxide aggregate (MTA) and Intermediate Restorative Material (IRM).

**MATERIALS AND METHODS:**The studied materials were prepared and placed in 24-wells plate. Human MG-63 osteoblasts were introduced to materials after their initial set. The supernatant fluid was collected after 1, 3, and 7 days and the level of interleukin-1β was measured by ELISA test. A microscopic exam was also performed to assess proliferation and viability of the cells. Kruskal-Wallis and Tukey tests were used for analysis.

**RESULTS:** ***T***here were significant higher levels of interleukin-1β in the gray and white MTA groups compared to IRM group (P<0.05). The difference in interleukin-1β secretion level between two mineral trioxide aggregate groups was not significant (P>0.05).Morphologic appearance of osteoblasts adjacent to gray and white MTA was similar to normal osteoblasts in all observation periods, however cells adjacent to IRM were round, signifying cytotoxicity of the adjacent material.

**CONCLUSION:**Human osteoblasts’ has a favorable biologic response to white and gray MTA compared to IRM.

## INTRODUCTION

**A**pproximately 6% of endodontic treatments are composed of endodontic apical surgery, perforation repair, and apexification treatment (1). The materials being used in these procedures are in direct contact with living tissues such as bone and connective tissue; in addition, the success rate of these treatments depends on regeneration of tissues. Different types of living cells are in direct contact with these materials among which fibroblasts, osteoblasts and cementoblasts are probably the most important ones. Accordingly, the ideal material in contact with living tissues should be biocompatible if not bioactive, unaffected by moisture, easy to manipulate, dimensionally stable and able to seal the root canal system (2).

Different dental materials have been introduced as root-end fillings including Cavit, composite resin, amalgam, glass ionomer cement, gutta-percha (both cold and injectable), gold foil, polycarboxylate cement, polyvinyl cement, various zinc oxide and eugenol-based cements (e.g. Super EBA and Intermediate Restorative Material (IRM)), mineral trioxide aggregate (MTA) and Dentin Bonding Agents (DBAs) (3). Even with efforts for introducing an ideal root-end filling, no material has been manufactured yet which includes all the assumed properties (4).

Comparing MTA, amalgam and Super EBA, Baek*et al.* found that MTA’shad most favorable periapical tissue response; this was due to cementum formation over MTA surface when being used in dog's teeth(5).

MTA was first introduce by Loma Linda University in the 1990s as a root-end filling and perforation repair material (6,7). Successful long-term results of this material in repairing root perforations have been reported (8). MTA (ProRoot; Dentsply, Tulsa Dental, OK, USA) is a powder consisting of fine hydrophilic particles. The main ingredients are tricalcium and dicalcium silicates, tri-calcium aluminate, tri-calcium oxide and silicate oxide. Bismuth oxide has been added as a radio pacifier(9).

It has been reported that MTA consists of calcium phosphate (10); however, energy dispersive analysis with X-ray could not detect the presence of phosphorus (11). The original formulation was gray in color and there were concerns regarding tooth discoloration (2). Recently, white MTA has been introduced which minimizes the problem of tooth and soft tissue discoloration (12). The difference between these two forms of MTA has been reported to be in the concentrations of aluminum, magnesium, and iron compounds (11). White MTA lacks the aluminoferrite phase that imparts the gray color (9). Although MTA has met most of the requirements of an ideal root-end filling material, its handling properties are less than ideal (10).

Several studies have been performed on root-end filling and perforation repair materials (13-16). These mainly focused on some characteristics of materials including biocompatibility, induction of hard tissue regeneration, and sealing ability. Biocompatibility of novel dental materials is established by *in vivo* tests in order to evaluate interactions between host and material (17). Most studies have been performed on gray MTA as white MTA is more recent. There is some conflicting data on the biocompatibility of gray and white MTA. This property has been investigated on gray MTA through cell expression and growth, subcutaneous and intra-osseous implantation, and direct contact with dental tissues *in-vivo* (18,19). Gray MTA is thought to be biocompatible, able to induce osteogenesis and cementogenesis, and also provide favorable seal for root and furcal perforations and apical foramina (20). The most commonly used methods for evaluation of cell proliferation are scanning electron microscopy (SEM) and enzyme assay. Enzyme assay measures the metabolic activity of cells which growover materials.

The main type of cytokine which is involved in pulp and periapical tissues inflammatory reactions is interleukin-1 (IL-1).This factor can influence the repair process positively and negatively (21). Therefore, IL-1 is considered as an indicator of biological status. In this *ex vivo*study we investigated the biological properties of gray MTA, white MTA and IRM by measuring the level of interleukin-1β secreted by human osteoblasts adjacent to the materials.

## MATERIALSAND METHODS

***Cell culture***

MG-63 osteoblasts from human osteosarcoma were obtained from National Cell Bank of Iran (NCBI, Pasteur Institute of Iran, Tehran). The cells were cultured in RPMI-1640 supplemented with 600µg/mL penicillin G, 300µg/mL strepto-mycin, 5µg/mL amphotericin B and 10% fetal bovine serum. The plates were incubated at 37^º^C in an atmosphere of 95% air and 5% carbon dioxide for7daystoachieveconfluence. 

***Preparation of materials***

The root-end filling materials used in the current study were gray and white MTA (Dentsply, Tulsa Dental, OK, USA) and IRM (Dentsply, Caulk, Milford, USA). The materials were mixed according to manufacturer’s instructions and placed in 24- wells plates (Falcon, MeylanCedaux, France) with the thickness of 1mm and a surface area of 1X1cm^2^; the materials were kept at 37^°^C and 100% humidity for 24hours to set completely. After 6 hours, 4X10^5^ MG-63 osteoblast cells were added to each well of the cell culture. There were 45 specimens (five specimens of each material for three time periods). After adding the osteoblasts, the supernatant fluids were collected after one, three and seven days; they were stored at -20^°^C also that the specimens were ready for assay.

**Figure1:**
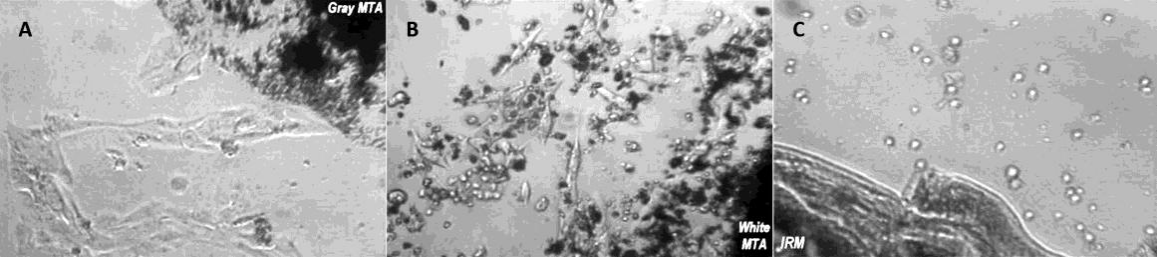
Light microscopic view of osteoblasts after 7days,* A)* gray MTA, *B)* white MTA, and *C)*IRM, (mag. ×40)

**Figure 2:**
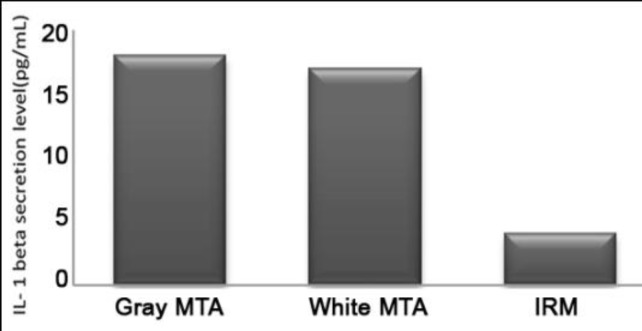
IL-1β  level secreted by human osteoblasts adjacent to different experimental materials(pg/mL)

Cell activity was measured in terms of cell secretion as determined by secretion of IL-1β using the Enzyme Linked Immunosorbent Assay (ELISA). Proliferation and viability of the cells treated by test materials were evaluated microscopically. Data were collected and analyzed by Kruskal-Wallis and the Tukey tests, avalue of P<0.05 was deemed significant.

## RESULTS

On the first day, light microscopy showed that osteoblasts adjacent to gray and white MTA show good attachment and morphological spread. Adjacent to IRM, all the cells were round and separated from the plate surface.

After three days cellular morphology adjacent to gray and white MTA show expansion and surface attachment demonstrating suitable tissue compatibility. Cells contacting with IRM seemed completely round and inactive.

After seven days microscopic view of the cells adjacent to white and gray MTA demonstrated active and vital osteoblasts confirming tissue compatibility (Figures1A, 1B), but cells adjacent to IRM were still round and inactive (Figure 1C).

In the evaluation of IL-1β secretion the Kruskal-Wallis test demonstrated that there were no significant differences in the mean levels of IL-1β secretion at different time intervals adjacent to each material (P>0.05).Kolmogorov-Smirnov test confirmed normal distribution of the data. The results are shown in Table 1 and Figure 2.Tukey test was used to compare the average levels of IL-1β secreted by the osteoblasts. There was no significant difference in the mean secretion level of IL-1β in presence of gray MTA and white MTA (P>0.05). However, the average amount of IL-1β in MTAs’ groups were significantly higher than IRM group (P<0.05).

## DISCUSSION

Reinforced zinc oxide and eugenol cements such as IRM and Super EBA are the most commonly used root-end filling materials (13,14). Previously, IRM has been widely used in root-end fillings and perforation repairs; however the toxicity of this material is proven. Accordingly, authors *e.g.*Koh*et al*. used IRM in their studies as the negative control (22).

Holland *et al*. showed that both gray and white MTA were biocompatible when implanted in rat connective tissues (23,24). Oviir*et al.* showed that cement oblasts grow significantly better on the surface of white MTA (25). In contrast, Pérez *et al.* showed that white MTA was not as biocompatible as the gray type (17). Different studies have shown that osteoblasts adjacent to this material morphologically alter and turn intoroundcells, indicating cytotoxicity (22).

Cell culture experiments are the method of choice for biocompatibility studies because they are simple, rapid, and cheap compared toother methods. A review recently declared that the two most commonly used methods for biocompatibility are SEM andenzyme assays(26). The main issue with SEM in cell culture studies was material’s reaction to preparation media. Enzyme assay was more reliable as it evades materials reactions(26).

**Table 1:** *IL*-1  levels (mean±SD) secreted by human osteoblasts (*Pico gram/milliliter*)adjacent to different test materials

**Material**	**Day 1**	**Day 3**	**Day 7**	**Mean**	**P-value**
**Gray MTA**	19.2±2.3	15.68±3.34	14.57±3.8	16.57	P>0.05
**White MTA**	15.85±0.92	14.76±1.8	16.03±3.4	15.59	
**IRM**	1.93±0.61	2.73±1.1	3.32±0.55	2.73	

Our study showed good IL-1β expression for both forms of MTA. The amount of IL-1β secreted by MG-63 osteoblasts adjacent to gray MTA and white MTA was significantly higher than that secreted by osteoblasts adjacent to IRM. There was no significant difference in the amount of IL-1β adjacent to gray MTA and white MTA.

MTA is known to cause an increase in IL-4 and IL-10 expression (27). Increase in IL-6 and IL-8, with no increase in level of IL-1α and IL-1β was demonstrated in the presence of MTA (28). Koh*et al*. showed a rise of both IL-1α and IL-1β together with IL-6 after the cells were in contact with the material for 6 days (22). They also showed no increase in cytokine release adjacent to IRM. MTA is also known to preferably induce alkaline phosphates expression and activity in fibroblasts of periodontal ligament and gingival cells (29).

In general, MTA elicited an inflammatory cytokine response. In contrast, no cytokine production was observed in one odd study, where the lack of cytokines was accompanied by cell lysis and proteinde naturating around MTA (18).

There is substantial evidence indicating theimportant role of cytokines in pulp and periapical diseases. The key cytokine involved in inflammatory pulp and periapical reactions is IL-1, secreted by a wide variety of cells. It is believed that all of the cells found in pulp and periapical lesions are capable of producing and secreting IL-1 (21). However, the most important cells that secrete IL-1 are monocytes, macrophages, endothelial cells, osteoclasts, osteoblasts, fibroblasts and PMNs (21).

IL-1α, IL-1β and tumor necrosis factor-alpha(TNF-α) promote bone destruction by osteoclasts. In humans, IL-1β has an OAF (osteoclast activating factor) role, indicating its high level of expression and its pharmacologic potential (30).It has also been demonstrated that IL-1 has an important role in acute inflammation.

On the other hand, it has been demonstrated that IL-1 has positive and negative roles in the repair process. It has also been found that proinflammatory cytokines have an important role in pulp protection by preventing the spread of infection (21). It is believed that large group of mediators such as Bone Morphologic Proteins (BMPs), Epidermal Growth Factor (EGF), IL-1 and IL-6 can accelerate infiltration, proliferation and differentiation of progenitor cells into osteoblasts (21).

Koh*et al.* compared the cytokines’ levels (IL-1α,IL-1β and IL-6)secreted by MG-63 osteoblasts adjacent to MTA and polymethylmethacrylate (PMA), the commonly used orthopedic cement. They demonstrated an increase in the secretory levels of these cytokines adjacent to gray MTA during the first 48 hours but the cells adjacent to PMA did not reveal any increase in the secretory levels of these cytokines (30).

The increase in the level of IL-1β in MTA containing specimens may demonstrate the activation of osteoblasts adjacent to gray MTA and white MTA.

## CONCLUSION

Human osteoblasts responded more actively adjacent to white and gray mineral trioxide aggregate compared to Intermediate Restorative Material, indicating the biocompatibility of these two materials.
